# Specificities of bipolar depression in psychiatric inpatients

**DOI:** 10.1192/j.eurpsy.2021.535

**Published:** 2021-08-13

**Authors:** M. Kacem, S. Khouadja, S. Brahim, I. Betbout, L. Zarrouk

**Affiliations:** 1 Department Of Psychiatry, University hospital of mahdia, chebba, Tunisia; 2 Psychiatry, University Hospital Of Mahdia, Mahdia, Tunisia; 3 Department Of Psychiatry, University Hospital of Mahdia, chebba, Tunisia; 4 Department Of Psychiatry, University Hospital Of Mahdia, Tunisia., Psychiatry, Mahdia, Tunisia

**Keywords:** Depression, bipolar, inpatients, specificities

## Abstract

**Introduction:**

Bipolar depression is not strictly clinically identical to unipolar depression.

**Objectives:**

To describe the clinical characteristics of patients with bipolar depression and to identify factors linked to bipolar depression.

**Methods:**

This is a cross-sectional, descriptive and comparative study carried out at the psychiatric department of the University Hospital of Mahdia. We have included 26 patients with bipolar depression and have compared them to 26 patients with unipolar depression. The data were collected from patients’ medical files. The analytical study has been made using Chi2 tests. The threshold of p<0.05 was considered as significant.

**Results:**

The mean age was 45 years. The majority of patients were male (61.5%) and unemployed (69.2%). Half of the patients were married. Alcohol consumption was found in 30.8% of cases. Family history of bipolar disorder and attempted suicide were present in 27% and 11.5% of cases respectively. A hospitalization number greater than or equal to 4 was found in 54% of cases. Personal history of suicide attempts was found in 46.2% of cases. At the psychiatric examination, psychomotor retardation, anxiety and psychotic and atypical characteristics were present in 73%, 31%, 42.3% and 7.7% of cases respectively. 46.2% of patients were treated with antidepressants in combination with a mood stabilizer. Antipsychotic treatment was combined in 80.8% of cases. A significant difference was noted for the number of hospitalizations, anxiety and antipsychotic treatment.

**Conclusions:**

An early distinction between bipolar and unipolar disorders is crucial for the treatment of both diseases.

